# A standardized extract of *Ginkgo biloba* prevents locomotion impairment induced by cassava juice in Wistar rats

**DOI:** 10.3389/fphar.2014.00213

**Published:** 2014-09-25

**Authors:** Eduardo Rivadeneyra-Domínguez, Alma Vázquez-Luna, Juan F. Rodríguez-Landa, Rafael Díaz-Sobac

**Affiliations:** ^1^Laboratorio de Farmacotoxicología, Facultad de Química Farmacéutica Biológica, Universidad VeracruzanaXalapa, México; ^2^Laboratorio de Biología y Química Molecular de Frutas, Instituto de Ciencias Básicas, Universidad VeracruzanaXalapa, México; ^3^Laboratorio de Neurofarmacologia, Instituto de Neuroetología, Universidad VeracruzanaXalapa, México

**Keywords:** *Manihot esculenta* Crantz, *Cassava*, neurotoxic, hippocampus, locomotor activity, *Ginkgo biloba*

## Abstract

The long-term consumption of cassava (*Manihot esculenta* Crantz) juice produce neurotoxic effects in the rat, characterized by an increased motor activity in the open field test and presence of uncoordinated swim (i.e., lateral swimming), in the swim test; which has been associated with damage in the hippocampus (CA1). On the other hand, flavonoids content in the *Ginkgo biloba* extract has been reported to produces neuroprotective effects at experimental level; therefore we hypothesized that *G. biloba* extract may prevents the motor alterations produced by cassava juice and reduce cellular damage in hippocampal neurons of the rat. In present study the effect of vehicle, cassava juice (linamarin, 0.30 mg/kg), *G. biloba* extract (dry extract, 160 mg/kg), and combination of treatment were evaluated in the open field and swim tests to identify locomotor and hippocampal alterations in adult male Wistar rats. All treatments were administered once per day, every 24 h, for 28 days, by oral rout. The effect was evaluated at 0, 7, 14, 21, and 28 days of treatment. The results show that cassava group from day 14 of treatment increase crossing and rearing in the open field test, as compared with the vehicle group; while in the swim test produces an uncoordinated swim characterized by the lateral swim. In this same group an increase in the number of damage neurons in the hippocampus (CA1) was identified. Interestingly, both behavioral and neuronal alterations produced by cassava juice administration were prevented by treatment with *G. biloba* extract. The results shown that *G. biloba* extract exert a protective effect against behavioral and neuronal damage associated with consumption of cassava juice in the rat. These effects are possibly related with flavonoid content in the *G. biloba* extract.

## INTRODUCTION

One of the main food for human beings are tubers and roots, due to its simplicity of production, easy storage of their derivates and the relative stability of the chemical compounds. The potato and cassava are the most important edible tubers because of its high carbohydrate content. Cassava root is usually consumed fresh, or processed as flour, some populations of Africa and America also consume leaves due to its medicinal properties (FAO and WHO, 2009; Rivadeneyra-Domínguez et al., 2013). However, cassava contains cyanogenic glycosides (linamarin and lotaustralin) which through hydrolysis produce cyanohydrin and glucose, and it is metabolized into acetone and hydrocyanic acid free. This metabolic process produces toxicity in the organism when it reaches concentrations above 100 μg (Ceballos and de la Cruz, 2002; Sornyotha et al., 2007).

Excessive consumption of cassava, an inappropriate processing and a low protein diet has been associated with neurodegenerative and neurological diseases such as lathyrism, hypothyroidism, Konzo, and Tropical Ataxic Neuropathy (TAN; Zaninovic, 2003; FAO and WHO, 2009). Furthermore, has been reported significant damage in brain structures involved in motor and cognitive integration including thalamic nuclei, piriform cortex, ventral nuclei, paraventricular area, and hippocampus, among others. In a previous study, Rivadeneyra-Domínguez et al. (2013) was reported that treated rats with cassava juice, in which linamarin was identified, shown alterations in motor incoordination (i.e., an increase in the lateral swimming) when they were evaluated to the swim test; which evidenced the neurotoxic effects associated probably with linamarin contained in the cassava juice.

On the other hand, *Ginkgo biloba* contains bioactive constituents mainly flavonoids (i.e., quercetin, kaempferol, and isorhamnetin), some diterpene trilactones such as ginkgolide A, B, and C sesquiterpene trilactonic, bilobalide, and proanthocyanidins (Koltermann et al., 2007), among others. Kaempferol and quercetin contained in *G. biloba* extracts are involved in the neuroprotective effects identified *in vivo* studies. Also it has been found that the neuroprotective effect of *G. biloba* extracts against excitotoxicity-induced damage associated with the overactivation of *N*-methyl-D-aspartate receptors, is associated with the high content of flavonoids and some sesquiterpenelactones. Additionally, flavonoids possess antioxidant activity, inhibiting the formation of hydroxyl and adriamicil radicals; reducing the lipid peroxidation (Pincemail et al., 1989; Filoteo et al., 1997; Xiao et al., 2006).

*Ginkgo biloba* extract also reduces neuroinflammation process; improves memory, learning, and cognitive function through a lower loss of muscarinic and α-adrenergic receptors. Additionally, *G. biloba* extracts increase the levels of choline acetyltransferase and somatostatin, in the cerebral cortex and some hippocampus regions (Tang et al., 2002; Spencer, 2009). Because *G. biloba* extract may exert neuroprotective effects in some brain structures, including hippocampus (Ying-Shan et al., 2000; Ahlemeyer and Krieglstein, 2003), and long-term consumption of cassava juice (*Manihot esculenta* Crantz) produces motor incoordination apparently associated with neurotoxic effects of linamarin contained in it, we hypothesized that the treatment with a *G. biloba* extract prevents motor incoordination in rats subjected to the swim test, and additionally reduces the neuronal damage in the hippocampus CA1 area.

## MATERIALS AND METHODS

### BIOLOGICAL MATERIAL

Cassava tubers (*M. esculenta* Crantz), free of agrochemicals, were collected from a traditional cultivation in the town of Defensa, Yecuatla county, Veracruz State, México (latitude19° 52′00″ N, longitude 96°47′00″ W) at an altitude 260 m above sea level (INEGI, 2010). The biological material was authenticated by professional staff who specialize in taxonomy from the Herbarium XAL at the Institute of Ecology A.C. (INECOL) in Xalapa, Veracruz, México.

### CASSAVA JUICE

Preparation cassava juice was performed according to procedures described in previous studies (Rivadeneyra-Domínguez et al., 2013). To avoid any degradation process, the cassava juice was freshly prepared every day before administration. Tubers were washed just after collection. They were peeled and cut into approximately 5 cm × 2 cm × 2 cm pieces. To standardize and facilitate the doses administered, the juice of the tuber was obtained with a juice extractor machine (Moulinex Model Centri III, Celaya, Guanajuato, México) and immediately administered *per os* to the rats.

### STANDARDIZED EXTRACT OF *Ginkgo biloba*

Dry standardized extract of *G. biloba* (Pharmaceutical presentation, VASODIL^®^, Laboratorios ALTANA Pharma, México) was used. It contains dry extract of *G. biloba* 40 mg/mL and 9.6 mg of standardized flavones glycosides calculated as quercetin and kaempferol. The used dose in present investigation was 160 mg/Kg of the rats’ weight.

### ANIMALS, EXPERIMENTAL GROUPS, AND TREATMENTS

Thirty-two adult male Wistar rats, weighing 250–300 g, were obtained from the vivarium of the Facultad de Medicina, Universidad Veracruzana, Xalapa, Veracruz, México. The rats were housed eight per cage in Plexiglas cages, with a 12 h/12 h light/dark cycle (lights on at 7:00 AM) and average room temperature of 25 ± 1°C. The animals had *ad libitum* access to water and food. All of the experimental procedures were performed according to the Guide for the Care and Use of Laboratory Animals (National Research Council, 1996) and the Norma Oficial Mexicana para el Uso y Cuidado de Animales de Laboratorio (Norma Oficial Mexicana, NOM-062-ZOO, 1999). All efforts were made to minimize animal discomfort during the course of this investigation.

A longitudinal study with four independent groups (*n* = 8 rats per group) was performed: a control group received vehicle of *G. biloba* extract plus vehicle of cassava juice, a second group received vehicle of *G. biloba* extract plus cassava juice, a third group received *G. biloba* extract plus vehicle of cassava juice, and the last group received *G. biloba* extract plus cassava juice. The vehicle or the extract of *G. biloba* was administered in an equivalent volume of 2 mL/Kg of the rats’ weights by oral rout (PO); containing 160 mg/2 mL of standardized extract of *G. biloba*. The vehicle or the juice of cassava was administered 30 min after administration of vehicle or extract of *G. biloba*, by PO in an equivalent volume of 2 mL/Kg of the rats’ weights, containing 0.30 mg of linamarin per 2 mL of cassava juice. All of the treatments were administered once per day, every 24 h, for 28 days through a displaceable sterilized intraesophageal polyethylene cannula (4 cm length, 1.0 mm diameter; S-54-HLCole-Parmer, Vernon Hills, IL, USA) coupled to a disposable syringe (Roger, Chalco, Estado de México, México). In the baseline session (day 0) without treatment, the rats were evaluated in the open field test and subsequently swim test. At the end of this first session, the treatments began. One hour after vehicle or cassava juice administration on days 7, 14, 21, and 28 of treatment, the rats were evaluated in the same behavioral tests. After the last swim test (day 28) the rats were perfused and the brains removed for subsequent histological analysis.

### BEHAVIORAL TESTS

All of the open field and swim test sessions were video-recorded. Two independent observers who were blinded to the treatments recorded the dependent variables using custom-designed software until they attained results with a confidence index of at least 95%.

### OPEN FIELD TEST

The rats were individually placed in an opaque Plexiglas cage (44 cm × 33 cm) with 20 cm high walls and the floor divided into 12 squares (11 cm × 11 cm). The number of crossings and rearings were recorded during a 5 min test. A crossing was scored when a rat passed from one square to another with its hindlimbs. A rearing was considered when the rat assumed a vertical posture, supported by its hindlimbs, with respect to the floor of the box. A crossing was interpreted as an indicator of motor activity, and rearing was used to determine alterations in motor coordination. The swim test was performed after the open field test.

### SWIM TEST

The rats were individually placed in a rectangular pool (26 cm × 29 cm × 50 cm) filled with water (25 ± 1°C), for 5 min. The depth of the water was adjusted so that the rat could touch the bottom of the pool with one or both of its hindlimbs and tail. At the beginning of the test, the rat was gently placed in one corner of the pool. The rats swam vigorously as soon as they contacted the water. None of the animals drowned. After this initial swimming behavior, the rats displayed lateral swimming as previously was described (Rivadeneyra-Domínguez et al., 2013). The dependent variable was the number of times that lateral swimming was displayed by the rat in this test. Lateral swimming is operationally defined as behavior in which the rat swims slowly on its side without maintaining its horizontal balance. During this behavior, the rat swims on its right or left side, with its head maintained horizontally. The hindlimbs remain extended and rigid, parallel to the water surface, for short periods of time. The hindlimbs are uncoordinatedly moved to achieve water displacement. One or both forelimbs remain retracted. After this lateral swimming, the rats eventually swim “normally” for short periods of time.

### HISTOLOGICAL ANALYSIS

#### Perfusion

At the end of behavioral tests (day 28 of treatment), the rats received an overdose of sodium pentobarbital (Sedalphorte^®^, Laboratorios Salud y bienestar animal, México) and were perfused transcardially with 200 mL of saline solution followed by 200 mL of 4% paraformaldehyde in 0.1 M phosphate buffer (Temperature 27°C, pH 8.3). The brains were removed and preserved in 20% formalin until histological analysis.

#### Dehydration brain

The brains were dehydrated in ethanol solutions (70, 80, 96, and 100%), cleared in xylene and embedded in paraffin. Serial coronal sections of 10 μm thick-sections at the level of the hippocampus: interaural 4.70 mm, Bregma 4.30 mm (Paxinos and Watson, 1998) were obtained with a vibratome.

#### Stains and preparation of brain slices

The brain slices containing the hippocampus were stained with the modified technique of hematoxylin–eosin and dried at room temperature (Lynch et al., 1982; Neira and Sedano, 2008). Posteriorly, the stained brain slices were mounted with Permount for analysis by light microscopy.

#### Quantification of damaged cells

Three fields of different slices that included the CA1 area of the right (Ap = -3.30 to -4.00 mm, *L* = 1.2 mm and *H* = 3.0 mm) or left (Ap = 3.30 to –4.00 mm, *L* = -1.2 mm, and *H* = 3.0 mm) hippocampus with respect to Bregma (Paxinos and Watson, 1998) were analyzed per rat from each group. The number of damaged cells in each experimental group was measured. Neuronal damage was considered when under optical microscope examination the presence of eosinophilic neurons (acidophilic) with nuclear changes characterized by the presence of eosinophilic cytosol and pyknotic or fragmented nuclei were identified; which are indicators of neuronal damage (Neira and Sedano, 2008).

#### Statistical analysis

The data from two behavioral tests were analyzed by two-way ANOVA, considering as the first factor treatment, and the second factor days of treatment. Significant differences (*p* ≤ 0.05) in the ANOVA were followed by Student-Newman-Keuls *post hoc* test. The data from the histological study were analyzed using Kruskal–Wallis one-way ANOVA on Ranks, followed by Tukey *post hoc* test. All data are expressed as the mean ± SE for each variable.

## RESULTS

### LOCOMOTOR ACTIVITY IN THE OPEN FIELD TEST

**Table 1** shows the results of crossing in the open field test in rats treated with vehicles, cassava juice, *G. biloba*, or combination of treatments (*G. biloba* plus cassava juice). The analysis of crossing show significant differences among treatments [*F*(3,140) = 19.778; *p* < 0.001], days of treatment [*F*(4,140) = 10.610; *p* < 0.001], and interaction between factors [*F*(12,140) = 2.344; *p* < 0.009]. The *post hoc* test showed that the vehicle and *G. biloba* treated groups reduce significantly the crossing throughout the experimental sessions. However, in the cassava treated group crossing was reduced in days 7 and 14 of treatment respect to the vehicle treated group, but it was recovered to basal values in days 21 and 28 of treatment. No significant differences were detected in the *G. biloba* plus cassava juice treated groups along of the experiment.

**Table 1:** Number of crossing in the open field test along of days of treatments.

Treatment	Basal	Day 7	Day 14	Day 21	Day 28
Control	44.5 ± 9.8	37.6 ± 8.7*	24.5 ± 3.57*	14.0 ± 4.49*	16.25 ± 2.5*
Cassava juice	44.5 ± 9.8	28.6 ± 4.1	29.1 ± 7.8	36.75 ± 5.1^+^	42 ± 2.1^+^
*Ginkgo biloba*	44.5 ± 9.8	17.3 ± 3.8*	19.6 ± 4.5*	8.0 ± 1.9*	11.0 ± 3.3*
Cassava juice + *Ginkgo biloba*	44.5 ± 9.8	14.1 ± 3.4	13.5 ± 2.4	11.0 ± 2.0	16.13 ± 2.3

The values represent the mean ± SE of eight rats. *p < 0.05 vs basal of the same group; +p < 0.05 vs. all groups in the same day of treatment (two-way ANOVA, post hoc test Student-Newman-Keuls).

**FIGURE 1:**
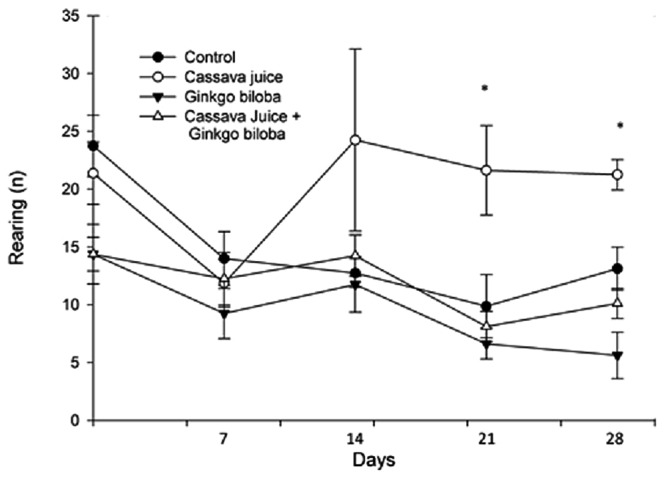
**Number of rearing in the open field test.** All experimental groups, except cassava juice group reduced rearing from day 14 of treatment respect to the basal values of the same group. *p < 0.05 vs all groups in the same day of treatment. (two-way ANOVA, *post hoc* test Student-Newman-Keuls)

The analysis of the rearing showed similar results to those obtained in crossing in the open field test; that behavior was affected by treatments [*F*(3,140) = 13.642; *p* < 0.001], days of treatment [*F*(4,140) = 4.712; *p* < 0.001], but not interaction between factors [*F*(12,140) = 1.540; *p* = 0.117] was identified. The *post hoc* test revealed that the control, *G. biloba* or *G. biloba* plus cassava juice groups reduced rearing from day 14 of the treatment until the end of the study, while the group receiving cassava juice treatment reduced rearing on day 7 days of treatment compared to the basal; but it was recovered to the basal values from day 14 of treatment (**Figure 1**).

### SWIM TEST

**FIGURE 2:**
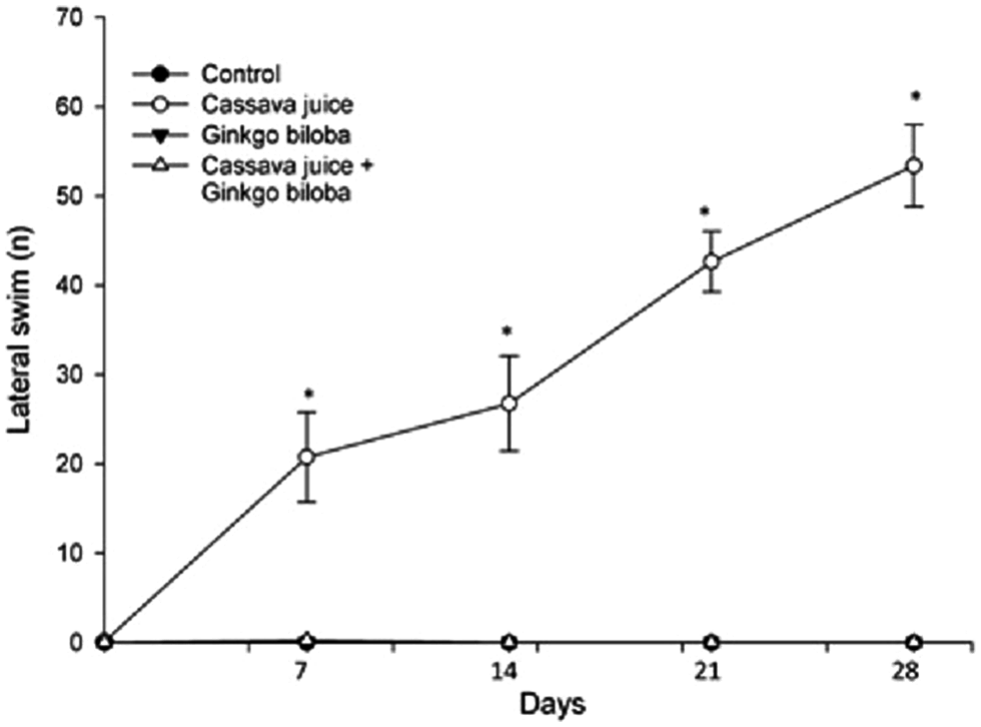
**Number of lateral swim.** This behavior was only detected in the cassava juice treated group, an effect prevented by simultaneous administration of *Ginkgo biloba* extract. *p < 0.05 vs basal and all groups in the same day of treatment. (two-way ANOVA, *post hoc* test Student-Newman-Keuls).

The analysis of the number of lateral swim showed significant differences considering treatments [*F*(3,149) = 237.654; *p* < 0.001], days of treatments [*F*(4,149) = 24.256; *p* < 0.001], and in the interaction of factors [*F*(12,149) = 24.332; *p* < 0.001]. The *post hoc* test revealed that rats control or treated with *G. biloba* did not presented lateral swimming along of the days of treatment. However, rats that consume cassava juice showed an increase in the number of lateral swim from day 7 of treatment and this effect being more accentuated at 28 day of treatment. Interestingly, this effect was no detected in the *G. biloba* plus cassava juice group (**Figure 2**).

### DAMAGED NEURONS IN CA1 AREA OF THE HIPPOCAMPUS

**FIGURE 3:**
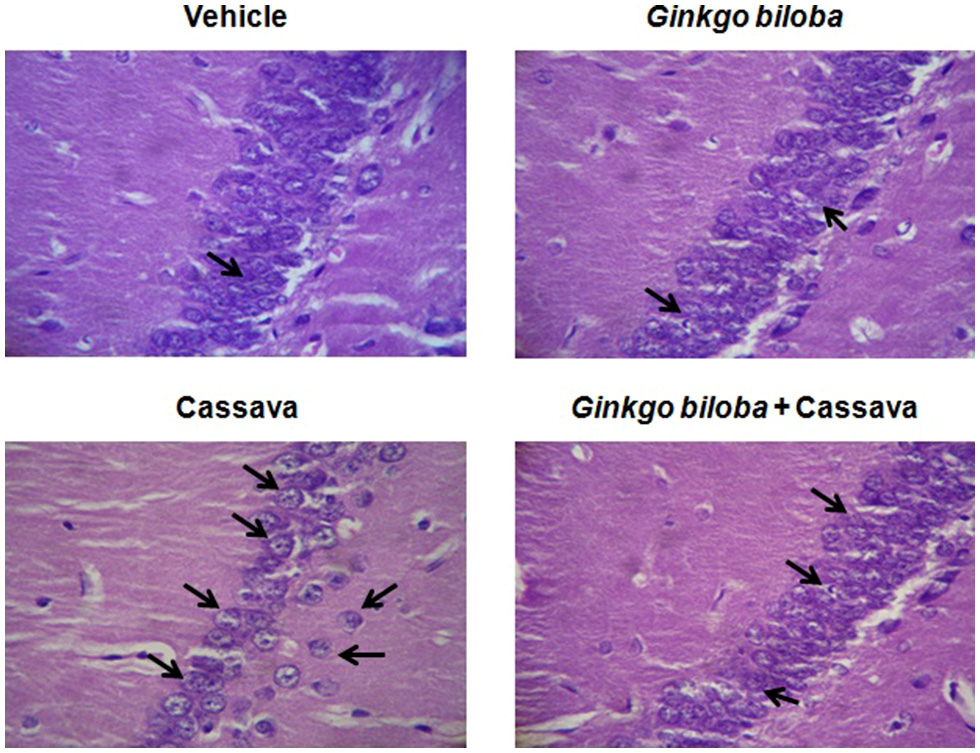
**Representative photography of hippocampus neurons (CA1) corresponding to each treatments.** The higher number of damage neurons was detected in cassava group, in which also a reduced number of neurons was observed compared with the other groups of treatment. Black arrow indicates hippocampus damaged neurons.

**Figure 3** show a representative photograph of CA1 hippocampus damage neurons in each treatment group. The analysis of the damage neurons in this area did not revealed significant differences among left or right side, therefore all neurons were including together as CA1 hippocampus area. The analysis of the number of damaged neurons in the CA1 area of hippocampus from different groups of treatment showed significant differences [*H*_(3)_ = 26.121; *p* < 0.001]. The *post hoc* test showed that cassava juice-treated group had the higher number (13.75 ± 1.90) of damaged neurons (*p* < 0.05), as compared with the others groups: control (0.50 ± 0.18), *G. biloba* (0.62 ± 0.18) and *G. biloba* plus cassava juice (3.00 ± 0.46). As showed the results *G. biloba* treatment prevents the neuronal damage associated with cassava juice treatment.

## DISCUSSION

The open field test is widely used to detect changes in locomotor activity, associated with experimental manipulations or drug administrations (i.e., hyperactivity, hypoactivity, or not changes associated with drug treatment). Repeated session in the open field test induces habituation in the animals, reducing crossing in subsequently session respect to basal session (Brudzynski and Krol, 1997). In the present study, the control, *G. biloba*, and *G. biloba* plus cassava juice groups, decreased locomotor activity in the open field along the experimental sessions, which is in accordance with previous reports (Spencer, 2009), considering the common habituation to this test after repeated sessions. Contrarily, the cassava juice group initially reduced crossing, but from day 14 of treatment crossing returned to basal values, which could be related with the neurotoxic effects produced by linamarin contained in the cassava juice as previously reported (Rivadeneyra-Domínguez et al., 2013). The fact that the *G. biloba plus* cassava juice group had a gradual reduction of crossing along of the experiment similarly to the control group, suggest a protective effect of *G. biloba* extract against the toxic effects produced by cassava juice identified by changes on the motor activity. Present result may be supported by the neuroprotective effects of *G. biloba* extracts previously reported (Pincemail et al., 1989; Filoteo et al., 1997; Xiao et al., 2006; Spencer, 2009). Respect to rearing in the open field also was gradually reduced along of experimental session as previous reports (Rivadeneyra-Domínguez et al., 2009, 2013; Saavedra et al., 2011), which is associated with an adaptive effect by the repetition of the test. However, rats treated with cassava juice on day 21 and 28 of treatment increasing this behavior respect to the other experimental groups. This effect support the motor alterations in the cassava treated group, and the protective effect of *G. biloba*, considering that group with the extract of this plant plus cassava juice did not increase rearing as cassava juice do it.

In present investigation, cassava juice treatment produced locomotor incoordination in the swim test, represented by lateral swimming. In a previous study was reported that long-term consumption of cassava juice (Rivadeneyra-Domínguez et al., 2013) or *Dioon spinulosum* seeds, induced motor alterations characterized by lateral swimming or rotational behavior in the swim test (Rivadeneyra-Domínguez et al., 2009) which was associated with the content of linamarin or neurotoxic methylazoxymethanol (MAM), respectively. Interestingly, when MAM was microinjected into the hippocampal (CA1) of the rat, a motor incoordination in the swim test was observed (Saavedra et al., 2011). These results, shown that hippocampus participate in the locomotor alterations induced by neurotoxic compounds of some plants.

Among the diseases associated with cassava consumption are TAN and Konzo, which are mainly characterized by paraparesis of the lower or upper extremities, ataxia, muscle weakness, and motor alterations (Tylleskär, 1994; Peterson et al., 1995; Zaninovic, 2003; FAO and WHO, 2009). Therefore it could be related with motor alterations observed in rats treated with cassava juice; which was prevented by coadministration of *G. biloba* extract in present investigation. We report a toxic effect on motor activity in rats treated with cassava juice and is suggested that this effect could be related with chemical compounds contained in the plant; however it is necessary to realize additional specific studies to identify or discard other toxics effect on muscle stiffness, respiratory, cardiovascular, digestive, metabolic systems and cancer that could occur under long-term cassava consumption.

Previous studies have reported that *G. biloba* extract reduces neuronal damage caused by intoxication with methyl parathion and preserve cellular organization of the cerebellum granular layer in the rat (Cárdenas et al., 2010). In present study cassava juice group had a high number of damaged cells in the hippocampus (CA1), which was not detected in *G. biloba* plus cassava juice group. Aforementioned, suggests a neuroprotective effect of *G. biloba* extract on hippocampal neurons, probably associated with a reduction of excitotoxicity on NMDA receptors; however, specific studies are required to clarify this propose. The effect of flavonoids on the brain and cellular function include neuroprotection, reduction of neuroinflammation and improve memory, learning, and cognitive function (Mu et al., 2007). Additionally, flavonoids contribute to permeability, nutrition, oxygen uptake, and ATP synthesis (Mahadevan and Park, 2008). Flavonoids from *G. biloba* extract produces antioxidant activity and neutralizes free oxygen and hydroxyl radicals increased during cerebral damage followed of the ischemia (Blecharz-Klin et al., 2009). Although present study did not explored the cellular mechanism involved in the hippocampal neuronal damage produced by cassava juice nor the apparent protective effect of the *G. biloba* extract, the results suggests that evaluated extract produces a neuroprotective effect in hippocampal cells against toxic compounds contained in cassava juice, which must be explored in future studies. However present results, suggest that hippocampus could participate in motor alterations detected in cassava treated group, which may be support by previous studies in which microinjection of neurotoxic chemical compounds isolated from cycads (Saavedra et al., 2011), produces motor alterations in the rat, similar to detected in cassava treated rats.

Finally, although the precise mechanisms underlying the protective effect of *G. biloba* extract has not been explored in this study, it may be associated with the flavonoid content in the *G. biloba* extract (Ahlemeyer and Krieglstein, 2003). In this way, there is evidence that flavonoids content in the *G. biloba* extract may prevent excitotoxicity induced by NMDA receptor stimulation. This is relevant, considering that some toxic compounds contained in cassava juice can contribute to the overactivation of NMDA receptors (Spencer, 2009), causing neurotoxic effect. Therefore, it suggests that *G. biloba* extract may have protective activity against toxic effects produced by chemical compounds contained in cassava juice and consequently prevents locomotion impairments in the rats.

## CONCLUSION

The long-term consumption of cassava juice produced locomotion impairment probably associated with neuronal damage in the hippocampus (CA1), which is attenuated by *G. biloba* extract in Wistar rats.

## Conflict of Interest Statement

The authors declare that the research was conducted in the absence of any commercial or financial relationships that could be construed as a potential conflict of interest.
